# Hyperglycemia Augments the Adipogenic Transdifferentiation Potential of Tenocytes and Is Alleviated by Cyclic Mechanical Stretch

**DOI:** 10.3390/ijms19010090

**Published:** 2017-12-28

**Authors:** Yu-Fu Wu, Yu-Ting Huang, Hsing-Kuo Wang, Chung-Chen Jane Yao, Jui-Sheng Sun, Yuan-Hung Chao

**Affiliations:** 1 School and Graduate Institute of Physical Therapy, College of Medicine, National Taiwan University, Taipei 10055, Taiwan; Jay5300@gmail.com (Y.-F.W.); r05428002@ntu.edu.tw (Y.-T.H.); hkwang@ntu.edu.tw (H.-K.W.); 2 Department of Kinesiology and Community Health, College of Applied Health Science, University of Illinois Urbana-Champaign, Champaign, IL 61801, USA; 3 Graduate Institute of Clinical Dentistry and Department of Dentistry, School of Dentistry, National Taiwan University, Taipei 10048, Taiwan; janeyao@ntu.edu.tw; 4 Dental Department, National Taiwan University Hospital, Taipei 10048, Taiwan; 5 Department of Orthopedic Surgery, College of Medicine, National Taiwan University, Taipei 10051, Taiwan; drjssun@ntuh.gov.tw; 6 Department of Orthopedic Surgery, National Taiwan University Hospital, Taipei 10002, Taiwan; 7 Center of Physical Therapy, National Taiwan University Hospital, Taipei 10048, Taiwan; 8 Rehabilitation Center, National Taiwan University Hospital Chu-Tung Branch, Hsinchu County 31064, Taiwan

**Keywords:** tendon, diabetes, glucose, mechanical stretch, differentiation, PPARγ

## Abstract

Diabetes mellitus is associated with damage to tendons, which may result from cellular dysfunction in response to a hyperglycemic environment. Tenocytes express diminished levels of tendon-associated genes under hyperglycemic conditions. In contrast, mechanical stretch enhances tenogenic differentiation. However, whether hyperglycemia increases the non-tenogenic differentiation potential of tenocytes and whether this can be mitigated by mechanical stretch remains elusive. We explored the in vitro effects of high glucose and mechanical stretch on rat primary tenocytes. Specifically, non-tenogenic gene expression, adipogenic potential, cell migration rate, filamentous actin expression, and the activation of signaling pathways were analyzed in tenocytes treated with high glucose, followed by the presence or absence of mechanical stretch. We analyzed tenocyte phenotype in vivo by immunohistochemistry using an STZ (streptozotocin)-induced long-term diabetic mouse model. High glucose-treated tenocytes expressed higher levels of the adipogenic transcription factors *PPARγ* and *C/EBPs*. PPARγ was also highly expressed in diabetic tendons. In addition, increased adipogenic differentiation and decreased cell migration induced by high glucose implicated a fibroblast-to-adipocyte phenotypic change. By applying mechanical stretch to tenocytes in high-glucose conditions, adipogenic differentiation was repressed, while cell motility was enhanced, and fibroblastic morphology and gene expression profiles were strengthened. In part, these effects resulted from a stretch-induced activation of ERK (extracellular signal-regulated kinases) and a concomitant inactivation of Akt. Our results show that mechanical stretch alleviates the augmented adipogenic transdifferentiation potential of high glucose-treated tenocytes and helps maintain their fibroblastic characteristics. The alterations induced by high glucose highlight possible pathological mechanisms for diabetic tendinopathy. Furthermore, the beneficial effects of mechanical stretch on tenocytes suggest that an appropriate physical load possesses therapeutic potential for diabetic tendinopathy.

## 1. Introduction

Diabetes mellitus (DM) is associated with a higher risk for tendon pathology [1,2,3]. Diabetic tendons are characterized by an altered microstructure and a compromised mechanical function [4]. As a result, the physical capacity and glycemic control of people with DM are weakened. Tenocytes, tendon-derived fibroblasts, maintain tendon function and homeostasis by expressing tendon-related genes. However, hyperglycemia, a primary cause of diabetic complications, suppresses the expression of crucial tenogenic factors in tenocytes [5]. Considering that the non-tenogenic differentiation of tendon stem cells is involved in tendon degeneration and that tenocytes also possess the intrinsic potential to transdifferentiate into non-tenocytes, fine control of tenocyte differentiation is necessary to maintain tendon homeostasis [6,7]. To date, whether a diabetic microenvironment drives the non-tenogenic transdifferentiation of tenocytes is still unclear.

The characteristics of degenerated tendons warrant the exploration of the non-tenogenic differentiation tendency of tenocytes. First, lipid deposits and adipocytes have been observed between tendon fibrils in aged or degenerated tendons, disrupting structural continuity and reducing collagen fiber strength [8,9]. Although no direct evidence, to our knowledge, links DM to intratendinous fat accumulation, insulin resistance has been associated with intramuscular lipid deposition [10,11]. In addition, calcification is one of the pathological characteristics associated with diabetic tendinopathy, and the erroneous differentiation of tendon stem cells into chondrocytes or osteoblasts is suspected of being involved in its pathogenesis [4,12]. Lastly, diabetic tendons are characterized by disorganized collagen deposition and extravagant scar formation during healing, which may result from excessive myofibroblast differentiation [13,14]. The existence of non-tenocytes in degenerated tendons can be a consequence of the non-tenogenic differentiation of resident tenocytes.

Physiological tensile loads are essential to maintain tendon homeostasis. Appropriate mechanical stretch in vitro can direct the tenogenic differentiation of pluripotent stem cells or promote the expression of tendon-related genes in tenocytes [7,15]. However, whether mechanical stretch can restore the tenogenic characteristics suppressed by hyperglycemia is unclear. In this study, we tested the hypotheses that (1) hyperglycemic conditions enhance the non-tenogenic differentiation potential of tenocytes and that (2) mechanical stretch can mitigate this adverse effect by strengthening the normal tenocyte phenotype. Using our in vitro hyperglycemic model, we found that high-glucose conditions increased the adipogenic differentiation of tenocytes, while exposure to mechanical stretch allowed tenocytes to retain a fibroblastic phenotype.

## 2. Results

### 2.1. High Glucose Elevates mRNA Expression of PPARγ and α-SMA

**Figure 1:**
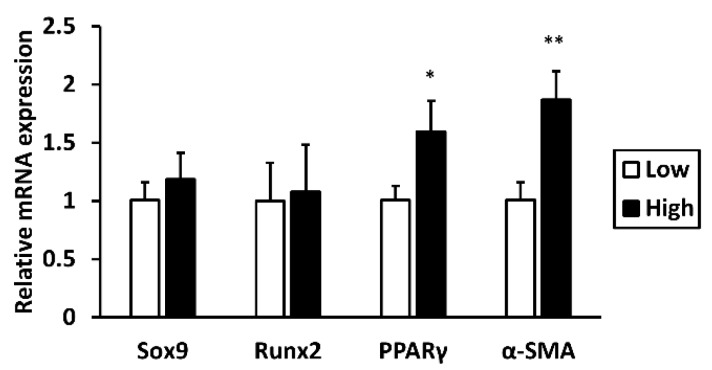
High glucose elevates mRNA expression of *PPARγ* and *α-SMA*. The mRNA expression levels of the non-tenogenic lineage markers *Sox9*, *Runx2*, *PPARγ*, and *α-SMA* were measured after tenocytes were cultured in high-glucose (25.5 mM) or low-glucose (5.5 mM) conditions for 1 week. Data are presented as the mean ± SD (*n* = 6). Statistical significance is shown as * *p* < 0.05 or ** *p* < 0.01 compared to the low-glucose control group. Experiments were performed as four biologically independent repeats.

To assess the transdifferentiation tendency of tenocytes into non-tenogenic lineages in high-glucose conditions, mRNA levels of the non-tenogenic gene markers *Sox9* (chondrogenic), *Runx2* (osteogenic), *PPARγ* (adipogenic), and *α-SMA* (myofibroblastic) were analyzed by qRT-PCR. In tenocytes cultured with high glucose for 1 week, *PPARγ*’s mRNA level was 1.60 ± 0.26-fold higher and *α-SMA*’s mRNA level was 1.87 ± 0.25-fold higher than the low-glucose group (Figure 1). Elevation in *PPARγ* and *α-SMA* expression was also observed in tenocytes cultured with high glucose for 2 weeks, suggesting that this upregulation is a long-lasting effect (Figure S1).

### 2.2. High-Glucose Conditions Increase the Adipogenic Transdifferentiation Potential of Tenocytes

**Figure 2:**
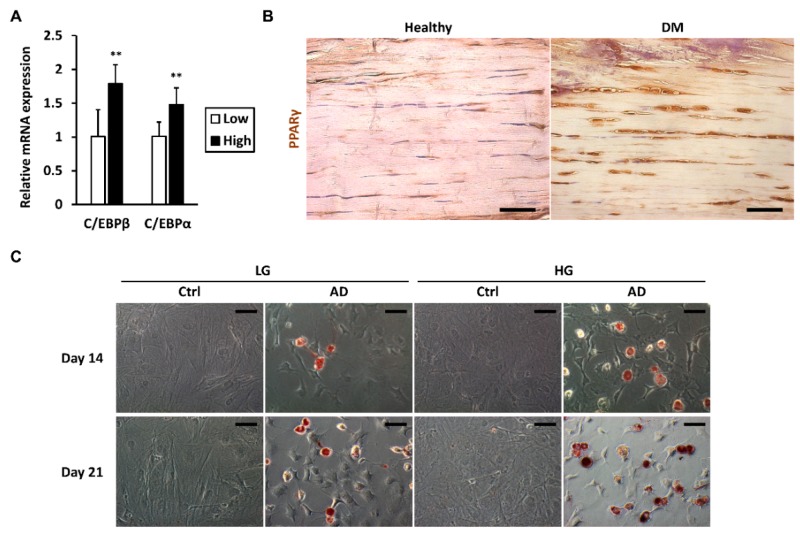
High glucose increases the adipogenic transdifferentiation potential of tenocytes. (**A**) The mRNA expression levels of the adipocyte markers *C/EBPβ* and *C/EBPα* were measured after tenocytes were cultured in high-glucose (HG) or low-glucose (LG) conditions for 1 week. Data are presented as the mean ± SD (*n* = 6). Statistical significance is shown as ** *p* < 0.01 compared to the low-glucose control group; (**B**) Tendons from STZ-induced type 1 diabetes mellitus (DM) mice (duration > 1 year) were analyzed by immunohistochemistry for PPARγ. Healthy: age-matched control mice without DM. Scale bar = 50 μm; (**C**) After pre-culturing in different glucose concentrations for 1 week, tenocytes were stimulated by adipogenic differentiation medium. Adipogenesis was examined using Oil Red O staining two and 3 weeks later. AD: adipogenic differentiation medium. Ctrl: culture medium. Scale bar = 50 μm.

Due to the observed upregulation of *PPARγ*, we further examined the expression of other adipogenic markers, *C/EBPβ* and *C/EBPα*. qRT-PCR analysis revealed that tenocytes cultured in high glucose expressed higher *C/EBPβ* and *C/EBPα* mRNA levels than did the low-glucose group (Figure 2A). To determine the presence of adipocyte-like tenocytes in diabetic tendons, Achilles tendons from mice with type 1 DM for over one year were analyzed by immunohistochemistry. Increased numbers of PPARγ-positive, rounded cells were found to reside in the diabetic tendons, aligning along the collagen fibrils, whereas the majority of tenocytes in healthy tendons were PPARγ-negative (Figure 2B). In accordance with the augmented expression of adipogenic markers, tenocytes in high-glucose conditions were more likely to transdifferentiate into adipocytes after long-term induction with adipogenic differentiation medium, confirmed by increased positively stained, rounded cells when stained with Oil Red O (Figure 2C).

### 2.3. High Glucose-Induced Increase in α-SMA Expression Does Not Indicate Myofibroblastic Differentiation

**Figure 3:**
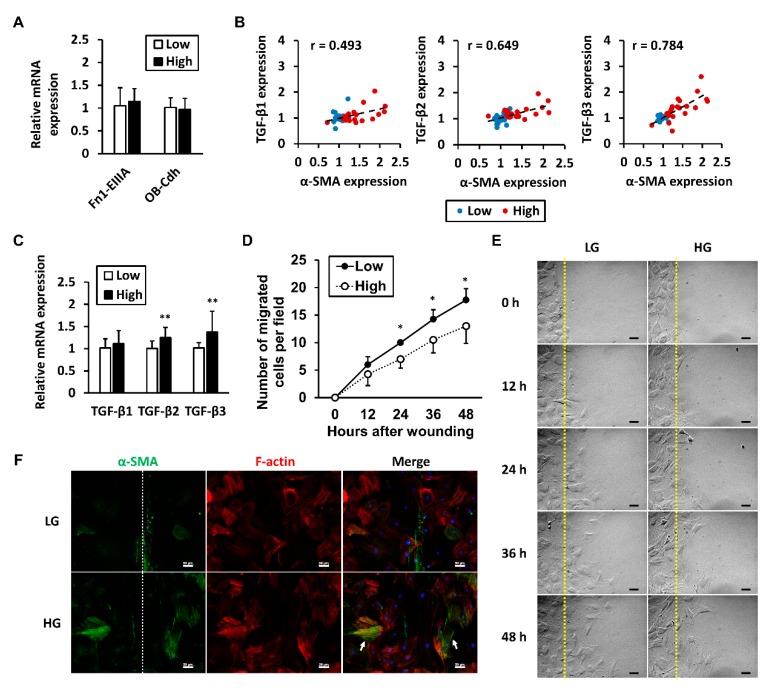
High glucose-induced increases in *α-SMA* expression do not indicate myofibroblastic differentiation. Tenocytes were cultured in high-glucose (HG) or low-glucose (LG) conditions for 1 week. (**A**) mRNA expression levels of the myofibroblast markers *Fn1-EIIIA* and *OB-cdh* were measured. Data are presented as the mean ± SD (*n* = 6); (**B**) Pearson’s correlation coefficients between mRNA levels of TGF-βs and *α-SMA* were calculated. Cell samples of both low- and high-glucose groups from four independent experiments (total *n* = 42) were included; (**C**) The mean ± SD of TGF-β mRNA levels in pooled samples are shown by group; (**D**) Cell migration rate is presented as the number of tenocytes that migrated into the wound at each time point (*n* = 3). Images of migrating cells are shown in (**E**), where the yellow dotted lines represent the outline of wounds; (**F**) F-actin and α-SMA (smooth muscle actin) were fluorescently labeled after wound closure, where white dotted lines represent the midline of wounds. No α-SMA-positive tenocytes migrated to the midline (arrows). Statistical significance is shown as * *p* < 0.05 or ** *p* < 0.01 compared to the low-glucose control group. Scale bar = 50 μm.

To examine the expression of other myofibroblastic markers besides *α-SMA*, the mRNA levels of ED-A domain-containing fibronectin (*Fn1-EIIIA*) and OB-Cadherin (*OB-Cdh*) were analyzed by qRT-PCR. Neither *Fn1-EIIIA* or *OB-Cdh* were significantly changed in response to high-glucose conditions (Figure 3A). Although excess TGF-β1 is well-known for its role in inducing myofibroblast differentiation and its association with human fibrotic diseases [16], each isoform of TGF-β can induce expression of *α-SMA* in fibroblasts in vitro [17]. A Pearson’s correlation analysis between mRNA levels of TGF-β and *α-SMA* showed that instead of *TGF-β1* (*r* = 0.493), *TGF-β3* (*r* = 0.784) had the highest correlation coefficient with *α-SMA*, with *TGF-β2* having the second highest (*r* = 0.649) (Figure 3B). Among the 42 individual samples, the mean differences between the high- and low-glucose groups were compared using student’s *t*-test. Significant differences were observed in *TGF-β2* and *TGF-β3* mRNA levels. Although both were higher in the high-glucose group, the mean differences were small. No significant difference was observed in *TGF-β1* (Figure 3C).

### 2.4. High Glucose Levels Reduce the Migration Rate of Tenocytes

The expression of filamentous α-SMA has been shown to retard cell migration, a crucial function for tenocytes [18]. An in vitro wound healing assay was used to assess the cell migration rate. In high-glucose conditions, tenocytes migrated more slowly than in the low-glucose group (Figure 3D,E). After wound closure, fluorescent labeling of the α-SMA and general F-actin demonstrated that the filamentous α-SMA in tenocytes was more prominent in the high-glucose group. However, in both groups, cells expressing filamentous α-SMA resided away from the middle line of the scratches (Figure 3F).

### 2.5. ERK and Akt Signaling Play Opposing Roles in Regulating PPARγ Expression

**Figure 4:**
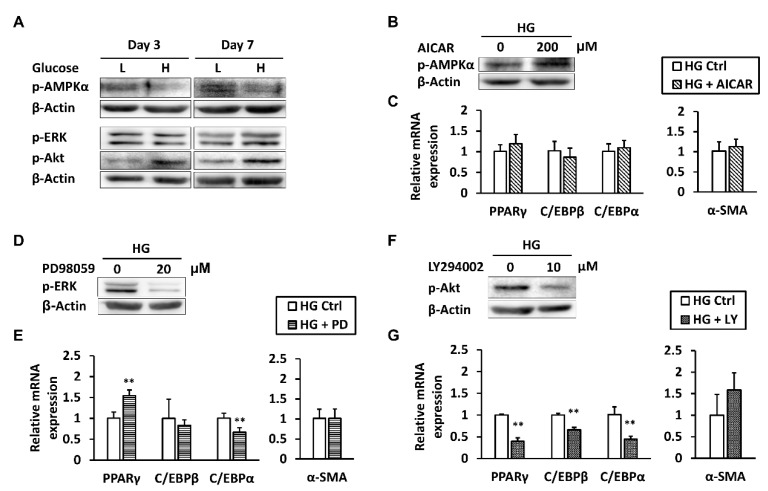
ERK and Akt signaling play opposing roles in regulating *PPARγ* expression. (**A**) Protein levels of p-AMPKα, p-ERK, and p-Akt in tenocytes cultured in low- or high-glucose conditions were measured. At day 3, after tenocytes in high-glucose conditions were treated with AICAR, PD98059, or LY294002 for 6 h; (**B**) p-AMPKα; (**D**) p-ERK; and (**F**) p-Akt protein levels were examined; and (**C**,**E**,**G**) the mRNA expression levels of adipogenic markers and *α-SMA* that were altered by high glucose were analyzed, respectively. Data are presented as the mean ± SD (*n* = 4). Statistical significance is shown as ** *p* < 0.01 compared to the DMSO-treated control group.

Our previous study demonstrated that high glucose-induced inactivation of AMPK signaling diminishes the expression of tenogenic markers [5]. On the other hand, MAPK/ERK and PI3K/Akt signaling pathways are involved in the enhancement of the adipogenic differentiation of mesenchymal stem cells in high-glucose conditions [19]. To identify which pathways were associated with the accumulation of adipogenic characteristics in high glucose-treated tenocytes, Western blot was used to analyze protein expression levels of phosphorylated AMPKα, ERK1/2, and Akt. At both day 3 and day 7 of high-glucose treatment, p-AMPKα level was suppressed, p-Akt was augmented, and p-ERK1/2 only showed a slight increase at day 7 (Figure 4A). At day 3, we manipulated these signaling pathways by administering the AMPK activator AICAR, the ERK inhibitor PD98059, or the Akt inhibitor LY294002 to the high-glucose cultured tenocytes. Then, expression of adipogenic gene markers as well as that of α-SMA, which were altered by high glucose, were examined by qRT-PCR. Although p-AMPKα level was increased by AICAR, none of the gene markers were significantly affected (Figure 4B). Inhibition of ERK1/2 signaling by PD98059 resulted in a 1.55-fold significant upregulation of *PPARγ* mRNA, while *C/EBPα* mRNA was downregulated (Figure 4C). Intriguingly, inhibition of PI3K/Akt signaling by LY294002 significantly suppressed all adipogenic gene markers. Specifically, *PPARγ* mRNA was downregulated by 40% compared to the control group (Figure 4D).

### 2.6. Mechanical Stretch Modulates ERK and Akt Signaling and Represses Adipogenic Differentiation

**Figure 5:**
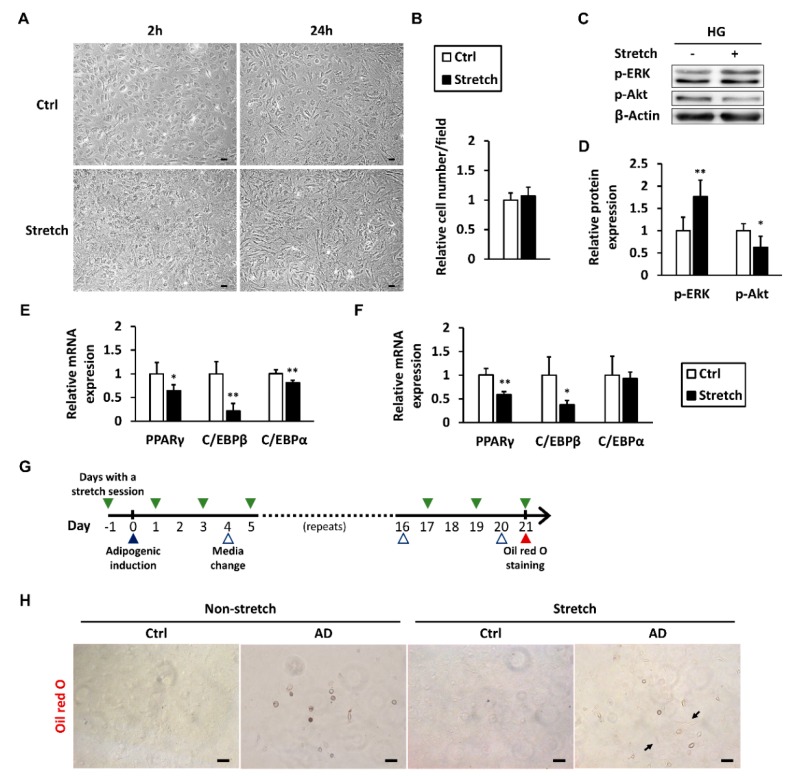
Mechanical stretch modulates ERK and Akt signaling and represses adipogenic differentiation. Tenocytes in high-glucose conditions were subjected to a 2-h mechanical stretch session. (**A**) Cell morphology was observed using a light microscope 2 and 24 h after the session began, scale bar = 50 μm; (**B**) Cell number was counted in three random visual fields from each of the three culture wells at the 24-h time point; 6 h after the stretch began, p-ERK and p-Akt in tenocytes were (**C**) immunoblotted and (**D**) quantified (*n* = 4); The mRNA expression of adipogenic markers was analyzed at (**E**) 2- and (**F**) 6-h time points; Data are presented as the mean ± SD (*n* = 4). Statistical significance is shown as * *p* < 0.05 or ** *p* < 0.01 compared to the non-stretched control group; (**G**) Timeline of the adipogenic differentiation protocol for tenocytes with regular exposure to mechanical stretch; (**H**) Adipogenesis was examined using Oil Red O staining on the 21st day of induction. Arrows indicate the stretched tenocytes retained their spindle shape. AD: adipogenic differentiation medium. Ctrl: culture medium. Scale bar = 50 μm.

We first assessed basic characteristics of stretched tenocytes. In high-glucose conditions, tenocytes treated with a 2-h session of mechanical stretch demonstrated a more spindle-shaped morphology (Figure 5A). Mechanical stretch did not significantly change the number of viable cells (Figure 5B). Expression levels of phosphorylated ERK and Akt, shown to be involved in the regulation of adipogenic gene expression, were next examined at 6 h after the stretch session began. Intriguingly, mechanical stretch significantly induced phosphorylation of ERK while simultaneously repressing that of Akt (Figure 5C,D). *PPARγ* and *C/EBPβ* mRNA expression levels were significantly reduced at both hours and 6 h after stretch began. C/EBPα, however, showed a slight decrease at the 2-h time point but no obvious change at the 6-h time point (Figure 5E,F). To explore the effect of mechanical stretch on adipogenic differentiation, a 2-h stretch session was administered every 2 days for 3 weeks, from the day before adipogenic induction to the day of Oil Red O staining (Figure 5G). After adipogenic induction, the non-stretched group showed more cells with rounded shape and red stain. In contrast to the non-stretched group, more of the stretched tenocytes retained their spindle shape (Figure 5H).

### 2.7. Mechanical Stretch Promotes Tenocyte Characteristics

**Figure 6:**
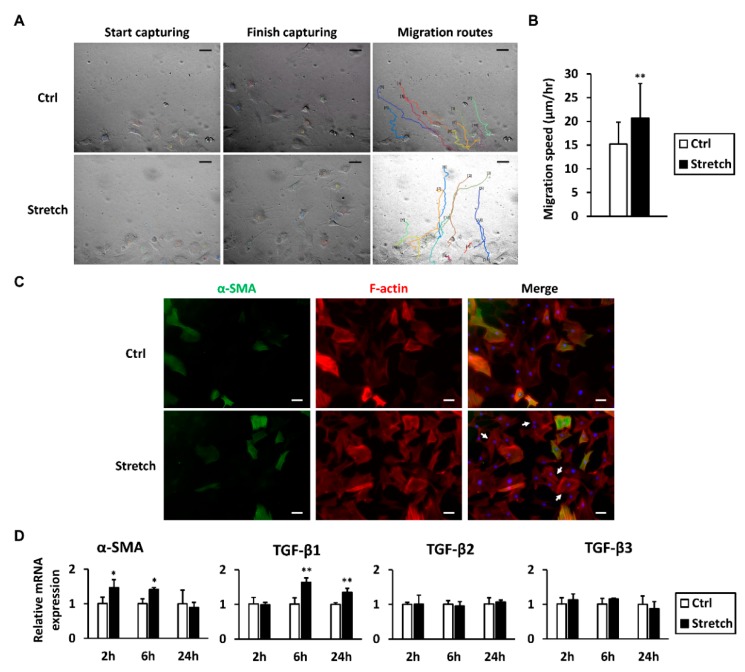
Mechanical stretch promotes tenocyte characteristics. (**A**) Motility of tenocytes subjected to mechanical stretch was analyzed using a wound healing assay. Wounds were created before stretch, and wound closure was monitored in real time up to 24 h. Migration routes were delineated using time-lapse videos. Each line represents the path of a different cell; (**B**) Migration speed of randomly chosen cells was calculated (*n* = 70); (**C**) F-actin and α-SMA were fluorescently labeled immediately after the stretch session. After stretch, F-actin in α-SMA-negative tenocytes were sharper than those in the non-stretched control group (arrows); (**D**) The mRNA expression levels of *α-SMA* and TGF-βs were measured 2, 6, and 24 h after stretch began (*n* = 4). Data are presented as the mean ± SD. Statistical significance is shown as * *p* < 0.05 or ** *p* < 0.01 compared to the non-stretched control group. Scale bar = 50 μm.

To examine tenocyte motility after mechanical stretch, a wound healing assay was performed followed by a migration route analysis from time-lapse videos. Mechanical stretch enhanced the migration rate of tenocytes in high-glucose conditions (Figure 6A,B). An immunofluorescence analysis revealed that, although α-SMA-positive tenocytes in both groups showed obvious F-actin fibers, α-SMA-negative tenocytes in the stretched group expressed higher levels of F-actin fibers, the structure of which were sharper than in the non-stretched group (Figure 6C). In the stretched group, *α-SMA* mRNA was transiently increased at 2 and 6 h after stretch began; however, it returned to baseline at the 24-h time point. Interestingly, *TGF-β1* mRNA was significantly elevated by mechanical stretch at 6 h, to a 1.64-fold level, and 24-h, to a 1.35-fold level, occurring later than the upregulation of *α-SMA*. Both *TGF-β2* and *TGF-β3* mRNA stayed at levels similar to the control group at each time point examined (Figure 6D).

## 3. Discussion

In summary, we used our previously published in vitro and in vivo hyperglycemic models to elucidate the deleterious effects of hyperglycemia on tenocyte differentiation [5]. Our in vitro data suggest that tenocytes cultured under high-glucose conditions augment the mRNA expression of the pivotal adipogenic transcription factors *PPARγ*, *C/EBPβ*, and *C/EBPα*, and enhance the efficiency of adipogenesis. Accordingly, tenocytes from diabetic tendons expressed higher levels of PPARγ proteins than the healthy tendons. These adipogenic effects of hyperglycemia were in part led by activation of the Akt pathway. Additionally, while expressing higher levels of α-SMA, high glucose-cultured tenocytes exhibited slower migration, implicating a reduction in tenocyte function. On the other hand, we demonstrated that mechanical stretch partially reversed the deleterious effects of hyperglycemia on tenocytes. Specifically, adipogenic gene expression and the tendency to transdifferentiate were diminished, while the fibroblastic morphology and motility of tenocytes were strengthened.

We previously illustrated that a 2-week, high-glucose, in vitro culture disrupted the expression of crucial tenogenic factors, including *Egr1*, implying that tenocytes in a long-term hyperglycemic condition exhibit limited ability to maintain their tenogenic cell fate [5]. Additionally, Lin et al. demonstrated that high glucose suppressed the expression of tendon-related markers in tendon-derived stem cells (TDSCs) in vitro [20]. On the other hand, Poulsen et al. found that oxidative stress enhanced the tenocyte phenotype in low-glucose conditions in vitro; however, in high-glucose conditions, tenocyte apoptosis was induced [21]. Both studies support our previous findings that tenocytes better maintain their tenogenic characteristics in low-glucose conditions than in high-glucose conditions. However, these two studies did not address whether high glucose induced non-tenogenic differentiation of tenocytes or TDSCs. To our knowledge, the present study is the first that uses long-term in vitro and in vivo diabetic models to investigate the non-tenogenic differentiation tendency of tenocytes.

The tendency of tenocytes to differentiate into any one of the mesenchymal lineages (e.g., adipocyte, chondrocyte, and osteoblast) or an undesired myofibroblast-like state was first screened by a marker gene expression analysis. Among the representative non-tenocyte gene markers, we found upregulation of *PPARγ* and *α-SMA* in high glucose-cultured tenocytes. Subsequently, we focused on investigating the effects of high-glucose conditions on either the adipogenic or myofibroblast transdifferentiation tendency of tenocytes.

The pivotal roles of *PPARγ*, *C/EBPβ*, and *C/EBPα* in adipogenic differentiation are well-documented. *PPARγ* is a master adipogenic regulator [22]. *PPARγ*-deficient mutant mice fail to form all kinds of fat tissues [23]. On the other hand, ectopic expression of *PPARγ* stimulates adipogenesis in *PPARγ^−/−^* mouse embryonic fibroblasts, suggesting its ability to induce adipogenic differentiation [24]. C/EBPβ is a determinant of adipogenic differentiation, and without treatment of adipogenesis-stimulating hormones, ectopic expression of *C/EBPβ* alone stimulated the terminal differentiation of fibroblast-like preadipocytes (3T3-L1) into adipocytes [25]. *C/EBPα* is also able to drive adipogenic differentiation in mouse fibroblastic cells [26]. Accumulation of *C/EBPα*, following a rapid increase of *C/EBPβ* expression during hormone-stimulated differentiation, suggests a later role for *C/EBPα* in adipogenesis [27]. During adipocyte maturation, *C/EBPα* coordinates with *PPARγ* to form a positive feedback loop in which they maintain each other’s expression, promoting the expression of various adipogenic genes [28,29]. Taken together, increases in the expression of these adipogenic markers indicate a higher tendency toward adipogenic differentiation.

High glucose has been shown to increase adipogenic differentiation in primary mesenchymal cells (MSCs) [19,30]. Chuang et al. found that mouse MSCs cultured in adipogenic induction medium with high glucose (25.5 mM) for 12 days expressed higher levels of *PPARγ* and exhibited higher adipogenesis efficiency compared to those with low glucose. A signaling protein analysis suggested that high glucose stimulates the activation of ERK and PI3K/Akt pathways during the induction period. Activation of both pathways was essential to the high glucose-induced increase in *PPARγ* expression and adipogenesis. In addition, this ERK- and PI3K/Akt-regulated adipogenesis was also found in bones from STZ-induced diabetic mice [19]. On the other hand, Zhang et al. demonstrated that high-glucose (25.5 mM) cultures induced adipogenic differentiation of rat osteoblasts, which was mediated by activated PI3K/Akt signaling [30]. In accordance with these two studies, our results suggest that high-glucose-cultured tenocytes not only augment the expression of adipogenic gene markers but also increase adipogenic differentiation. We found that activation of the PI3K/Akt pathway plays an essential role in maintaining the expression of the adipogenic factors, whereas ERK activation downregulates *PPARγ* expression. Furthermore, although we did not examine lipid accumulation in diabetic tendons, more PPARγ-positive tenocytes were found to reside in diabetic tendons from our long-term STZ-induced DM mouse model, compared to control healthy tendons.

The expression of filamentous α-SMA in fibroblasts represents an active myofibroblastic phenotype, rendering a contractile feature to the cell. These αSMA-expressing myofibroblasts play a crucial role in tissue repair through extracellular matrix synthesis and wound contraction regulated by TGF-β [31]. However, when assessing myofibroblast differentiation, other markers should also be considered. For instance, in healing wounds, ED-A domain-containing fibronectin is necessary for TGF-β-induced fibroblast-to-myofibroblast transition, and OB-cadherin is essential to enhance α-SMA-mediated contractile activity [32,33]. We found that high glucose did not change the expression of ED-A fibronectin or OB-cadherin. In addition, although excessive TGF-β1 is considered to cause scar fibrosis in healing tendons via inducing myofibroblast differentiation of tenocytes [34], we showed that, among the three isoforms, TGF-β1 was the least likely to mediate the high glucose-induced increase in α-SMA. Even though there were stronger positive correlations between *TGF-β2/TGF-β3* and *α-SMA* expression levels, these two genes showed only small differences between high- and low-glucose groups, suggesting that TGF-βs play a minor role in regulating *α-SMA* expression in our hyperglycemic model. Taken together, these findings warrant further investigation into myofibroblast differentiation of tenocytes in high-glucose conditions. Another hypothesis is that tenocytes in high-glucose conditions tend to dedifferentiate into *PPARγ+/α-SMA+* adipose progenitor cells, which have been found to reside in a vascular niche in white adipose tissues and contribute to adult adipogenesis [35]. Supporting evidence for these assertions from our study in high-glucose conditions include the following: (i) the expression levels of *PPARγ* and *α-SMA* were simultaneously increased; and (ii) the adipogenic differentiation potential of tenocytes was enhanced.

In the present study, the mechanical stretch protocol we utilized was established based on previous studies from other groups that demonstrated that 8% strain at a 1 Hz rate is the best stimulus to induce tenogenic differentiation [36,37]. In addition, to more closely mimic a real exercise experience, we set the total stretch time to 2 h per session. We demonstrated that mechanical stretch reversed the adverse effects induced by high glucose. Mechanical stretch not only repressed adipogenic characteristics but also reinforced the fibroblastic phenotype. Specifically, the adipogenic markers *PPARγ* and *C/EBPβ* were suppressed by stretch, partly via inhibition of Akt and activation of ERK. Mechanical stretch has been shown to inhibit *PPARγ* expression and adipogenic differentiation of 3T3-L1 cells by activating ERK [38]. Nevertheless, to our knowledge, this is the first study showing that mechanical stretch inhibits the essential pro-adipogenic Akt signal [39]. Moreover, Lamers et al. demonstrated that fibroblast migration was decreased by high-glucose conditions via a mechano-sensitive pathway [40]. Similarly, our data suggest that tenocyte motility was reduced by high glucose. After mechanical stretch, cell morphology was elongated, F-actin formation was enhanced in α-SMA-negative tenocytes, and tenocyte migration rate was increased. These findings imply that stretch may activate certain mechanical signaling pathways to reinforce tenocyte motility. In fact, Goldyn et al. demonstrated that after fibroblasts were exposed to an 8%-strain cyclic stretch at a 1 Hz rate, the Rho activity was increased, F-actin was re-oriented, and the rate of cell migration was enhanced [41].

Several limitations of this study should be considered. First, since our in vitro model only showed the influences of high glucose within 1 week, it may not be sufficient to explain the effects of chronic hyperglycemic microenvironment on tenocytes in vivo. Moreover, our in vivo evidence only demonstrated the existence of adipocyte-like tenocytes, exhibiting high PPARγ levels, in diabetic tendons. However, neither the adipogenesis capacity of these cells or intratendinous lipid deposition was examined, thus the in vivo findings are insufficient to fully complement our in vitro results.

## 4. Materials and Methods

### 4.1. Primary Tenocyte Isolation and Culture

Achilles tendons were obtained from 6-week-old, male, healthy Sprague-Dawley rats, cut into 2–3 mm^3^ pieces, and placed into six-well culture plates. Cells were propagated in Dulbecco’s modified Eagle’s medium (DMEM) with 10% fetal bovine serum (FBS) (Gibco) in a humidified 5% CO_2_ incubator at 37 °C. After attaining a subconfluent state (10–14 days later), cells were trypsinized and subcultured in 100 mm culture plates and medium was changed every three days. Cells from passage 3 to 8 were used in this study. For experiments, trypsinized cells were seeded onto either six-well culture plates or type I collagen-coated, flexible six-well culture plates (Bioflex; Flexcell International Corporation, Hillsborough, NC, USA) at 10^5^ cells per well, incubated in either high- (25 mM) or low-glucose (5.5 mM) DMEM, with FBS concentration reduced to 2% to avoid overgrowth. All study procedures received approval from the Institutional Animal Care and Use Committee of the National Taiwan University College of Medicine and College of Public Health (IACUC Approval No.: 20150362 and 20160402), and the Guide for the Care and Use of Laboratory Animals (Chinese-Taipei Society of Laboratory Animal Science, Taipei, Taiwan).

### 4.2. RNA Extraction and RT-qPCR

RNA extraction and a real time quantitative polymerase chain reaction (RT-qPCR) analysis were executed as previously described [5]. Briefly, total RNA was isolated from six-well, plate-cultured tenocytes using TRI Reagent^®^ and a Direct-zol^™^ RNA Kit (Zymo Research, Irvine, CA, USA). Total RNA was reverse transcribed into cDNA with PrimeScript^™^ RT reagent Kit (Takara, Kyoto, Japan). RT-qPCR was performed using the ABI 7900 system (Applied Biosystems, Foster, CA, USA), with a SensiFAST™ SYBR^®^ Hi-ROX Kit (Bioline, London, UK). Each reaction contained 50 ng of cDNA. Primer sequences are listed in Table 1. Relative gene expression level was calculated by the 2^(−ΔΔ*C*t)^ method using HPRT1 and GAPDH as internal controls.

### 4.3. Adipogenic Differentiation

Tenocytes were pre-incubated in low (5.5 mM) or high (25 mM) glucose medium with 2% FBS for 1 week. Medium was then changed into rat adipocyte differentiation medium (Cell Applications) and medium was changed every four days. Cells were analyzed by Oil Red O staining at 14 and 21 days post-adipogenesis induction.

### 4.4. Oil Red O Staining

Cells were washed twice with phosphate buffered saline (PBS), fixed with 4% para-formaldehyde for 1 h, and rinsed with distilled water. Cells were soaked with 60% isopropanol and then stained with the Oil Red O/60% isopropanol solution for 7 min. Cells were promptly rinsed once with 30% isopropanol and washed with distilled water thoroughly. Cytoplasmic lipid droplets appeared red under a light microscopy examination.

### 4.5. Wound-Healing Migration Assay

After pre-incubation in low- or high-glucose medium for 1 week, a scratch was created in each well using P1000 pipette tips. A real-time Cell Culture Monitoring system (ASTEC CCM-MULTI) was used to observe migrating cells. Cell migration rate was analyzed by manually counting the number of migrated cells or by using a cell tracking system to delineate the cell migration routes [42].

### 4.6. Immunofluorescence

Tenocytes cultured on coverslips or type I collagen pre-coated membranes were fixed with 4% paraformaldehyde for 10 min and permeabilized with 0.3% Triton X-100 in PBS for 15 min at room temperature. After applying blocking buffer containing 5% FBS and 0.3% Triton X-100, cells were incubated first with anti-α-smooth muscle actin antibody (1:100, Abcam, Cambridge, UK, ab5694), and then with Goat Anti-Rabbit Alexa Fluor^®^ 488 secondary antibody (1:200, Abcam, Cambridge, UK, ab150077). Lastly, to stain F-actin, cells were incubated with tetramethylrhodamine isothiocyanate (TRITC)-conjugated phalloidin (1:250, ThermoFisher, Waltham, MA, USA, A12380). Stained cells were mounted using DAPI-containing mounting reagent (Abcam, Cambridge, UK, ab104139), and images were collected using a fluorescence microscope (Zeiss AXIOVERT 200 M).

### 4.7. Western Blotting

Tenocytes were lysed using RIPA buffer (Millipore, Burlington, MA, USA). Lysates were centrifuged, and the supernatant was collected. An equal amount of protein (30 μg) per gel lane was loaded on 8% polyacrylamide gels, separated by SDS-PAGE, and transferred onto PVDF membranes. The membranes were blocked with TBST, pH 7.5, containing 5% BSA and 0.05% Tween 20, for 1.5 h at room temperature. For immunolabeling, membranes were incubated with monoclonal primary antibodies specific for phospho-ERK (1:2000, Cell Signaling, Danvers, MA, USA, 4370S), phospho-Akt (1:1000, Cell Signaling, Danvers, MA, USA, 9271S), phospho-AMPKα (1:500, CusAb, CSB-PA477914), or β-Actin (1:3500, CusAb, CSB-PA000350) at 4 °C overnight, and then with HRP-conjugated secondary antibodies (1:5000, Jackson ImmunoResearch, West Grove, PA, USA, 111-035-003) at room temperature for 1 h. Immunolabeled proteins were visualized by Chemiluminescent HRP Substrate (Millipore, Burlington, MA, USA), imaged using ImageQuant LAS 4000, and quantified with ImageJ software (NIH, Bethesda, MD, USA).

### 4.8. In Vitro Mechanical Stretch

Biaxial cyclic stretch was applied on in vitro cultured tenocytes using a BioFlex strain unit (FX5000, Flexcell International Corporation, Hillsborough, NC, USA). The stretching parameter was set to 1 Hz rate, sine wave, and 8% strain. During the stretch session, tenocytes were cyclically stretched for 2 h. Previous studies from other groups have demonstrated that 8% strain at a 1 Hz rate is the best stimulus to induce tenogenic differentiation [36,37]. For the 3-week adipogenic induction experiment, this 2-h stretch session was performed every other day, beginning the day before adipogenic induction. Cells cultured in the same environment without stretching served as controls.

### 4.9. Diabetic Mouse Model and Immunohistochemistry

The establishment of the STZ-induced type 1 DM mouse model and the collection of the tendon samples were described in our previous study [5]. DM mice were kindly provided by Dr. Hong-Wei Chang of the Department of Internal Medicine at the National Taiwan University Hospital. Briefly, one year after DM induction, mice were sacrificed by CO_2_ asphyxiation, and total Achilles tendons (from myotendinous junction to calcaneus bone) were carefully excised and collected. Tissues were fixed in 4% paraformaldehyde, embedded in paraffin, and sectioned. Tissue sections were then stained with hematoxylin and immuno-labeled with anti-PPARγ antibody (Abcam, Cambridge, UK; ab209350) as the primary antibody. Images were obtained with a light microscope system.

### 4.10. Statistical Analysis

All data are presented as the mean ± standard deviation (SD). Mean differences were statistically evaluated by two-tailed Student’s *t*-test. Correlation between two data sets was calculated using Pearson’s correlation analysis.

## 5. Conclusions

We posit that mechanical stretch improves tenocyte characteristics via reversing the fibroblast-to-adipocyte phenotypic transition induced by high glucose. To our knowledge, this is the first study that sheds light on the signaling crosstalk between metabolic and mechanical effects on tenocytes. Our findings warrant future studies in the application of exercise therapy to an in vivo diabetic animal model to ultimately identify and optimize the molecular and cellular benefits of exercise prescription to diabetic patients with tendinopathy.

## Figures and Tables

**Table 1:** RT-qPCR Primer Sequences.

Oligo Name (Forward: F; Reverse: R)	Sequence (5′ to 3′)
***HPRT1***_F	AAGCTTGCTGGTGAAAAGGA
***HPRT1***_R	CCGCTGTCTTTTAGGCTTTG
***GAPDH***_F	GTGGACCTCATGGCCTACAT
***GAPDH***_R	GGATGGAATTGTGAGGGAGA
***Sox9***_F	CTGCGACCTCAGAAGGAAAG
***Sox9***_R	CGCTGGTATTCAGGGAGGTA
***Runx2***_F	AAGTGCGGTGCAAACTTTCT
***Runx2***_R	AGGCTGTTTGACGCCATAGT
***PPARγ***_F	CATTTTTCAAGGGTGCCAGT
***PPARγ***_R	GAGGCCAGCATGGTGTAGAT
***α-SMA***_F	CTGCCCGGAGACCCTCTTC
***α-SMA***_R	GCGAGGGCTGTGATCTCCTT
***C/EBPβ***_F	CAAGCTGAGCGACGAGTACA
***C/EBPβ***_R	CAGCTGCTCCACCTTCTTCT
***C/EBPα***_F	TTACAACAGGCCAGGTTTCC
***C/EBPα***_R	CAGTACACACAAGGCGGATG
***Fn1-EIIIA***_F	TTGCCTGGGAAAGCCCACAG
***Fn1-EIIIA***_R	CTCTCCATGCCACCGTGCAA
***OB-Cdh***_F	GGCACTGTGGTTGGGAGAGT
***OB-Cdh***_R	AGCCAGGCAGTTTCTTCCCT
***TGF-β1***_F	ATACGCCTGAGTGGCTGTCT
***TGF-β1***_R	TGGGACTGATCCCATTGATT
***TGF-β2***_F	CTGGAACCACTGACCATCCT
***TGF-β2***_R	AACTCCCTCACGTCACGAAC
***TGF-β3***_F	GCGTCTCAAGAAGCAGAAGG
***TGF-β3***_R	GCAGTTCTCCTCCAAGTTGC

## Supplementary Materials

Supplementary materials can be found at www.mdpi.com/1422-0067/19/1/90/s1.

## Abbreviations

PPAR	Peroxisome proliferator-activated receptor γ
ERK	Extracellular-signal-regulated kinase
Runx2	Runt-related transcription factor 2
α-SMA	α-smooth muscle actin
C/EBP	CCAAT/enhancer binding proteins
Fn1-EIIIA	ED-A domain-containing fibronectin
OB-Cdh	OB-Cadherin
TGF-β	Transforming growth factor-β
AMPK	AMP-activated protein kinase
MAPK	Mitogen-activated protein kinase
PI3K	Phosphoinositide 3-kinase
AICAR	5-Aminoimidazole-4-carboxamide-1-β-4-ribofuranoside
