# Supplementary material for: Hyperglycemia Augments the Adipogenic Transdifferentiation Potential of Tenocytes and Is Alleviated by Cyclic Mechanical Stretch
Source: Int J Mol Sci. 2017 Dec 28;19(1):90. doi: 10.3390/ijms19010090 (PMC5796040; doi:10.3390/ijms19010090)
Supplement: Supplementary file 1 [file ijms-19-00090-s001.pdf]

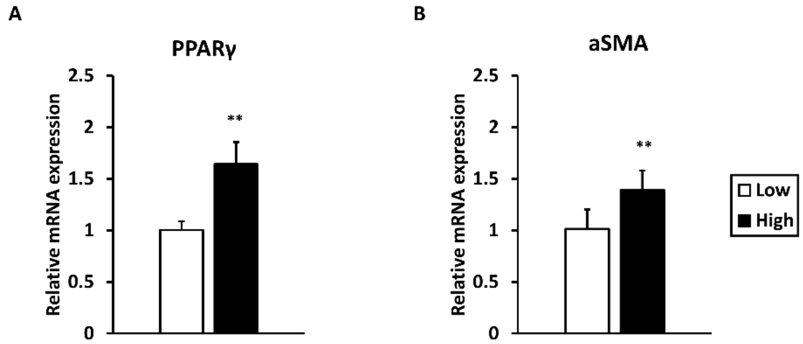

Supplementary Figure S1. High glucose keeps elevation in the mRNA expression of *PPARγ* and *α-SMA* to the second week. The mRNA expression levels of (**A**) *PPARγ* and (**B**) *α-SMA* were measured after tenocytes were cultured in high-glucose (25.5mM) or low-glucose (5.5mM) condition for two weeks. Data are presented as mean ± SD (n = 6). Statistical significances are shown as *$p<0.05$ or **$p<0.01$ when compared to low-glucose control group. Experiment was performed in four biologically independent repeats.
